# Supplementary material for: Analysis of Antimicrobial Resistance Genes (ARGs) in Enterobacterales and A. baumannii Clinical Strains Colonizing a Single Italian Patient
Source: Antibiotics (Basel). 2023 Feb 23;12(3):439. doi: 10.3390/antibiotics12030439 (PMC10044399; doi:10.3390/antibiotics12030439)
Supplement: Supplementary file 1 [file antibiotics-12-00439-s001.zip › antibiotics-2183416-supplementary.pdf]

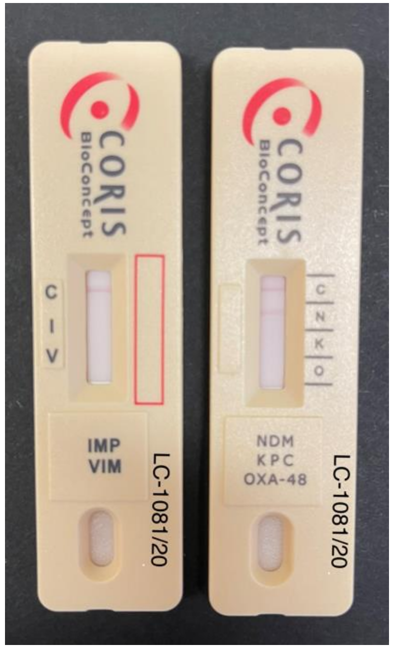

Proteus mirabilis RS

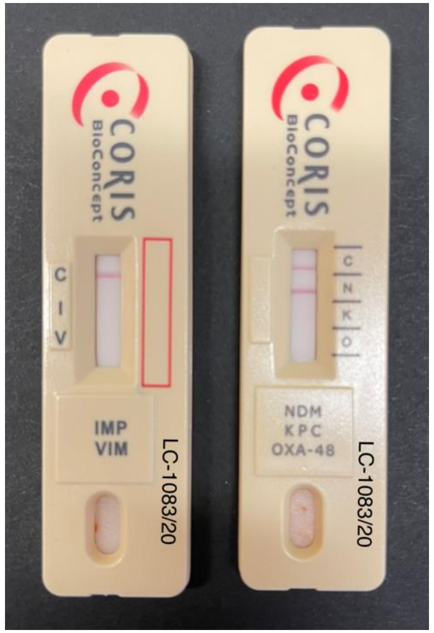

Klebsiella pneumoniae RS

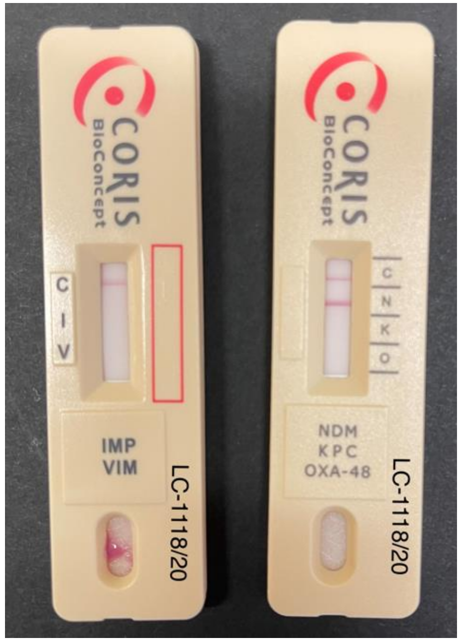

Enterobacter cloacae SW

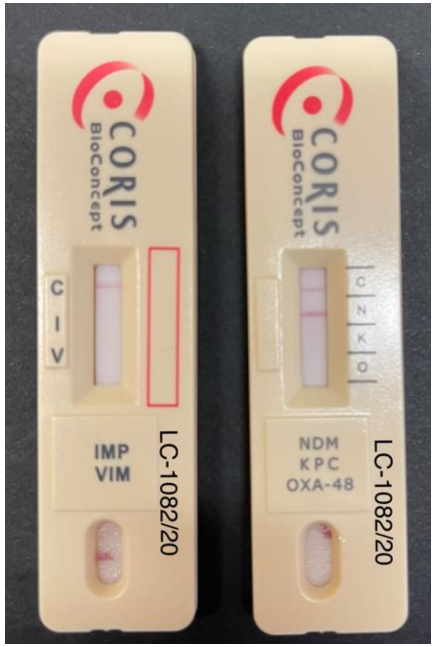

Enterobacter cloacae RS

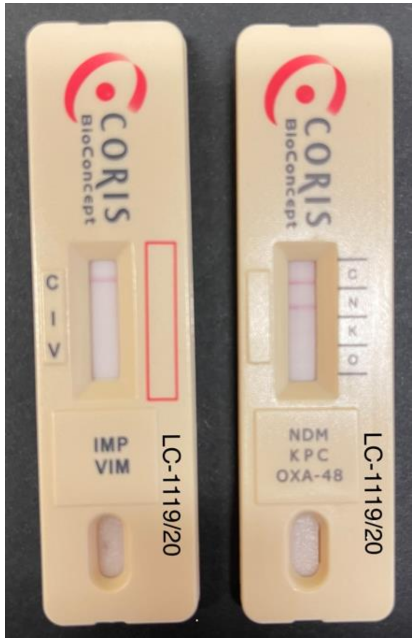

Proteus mirabilis SW

Figure S1. (a) Immunocromatographic test RESIST-4 O.K.N.V.I. (Coris BioConcept) to detect the presence of metallo-β-lactamases (MBLs)

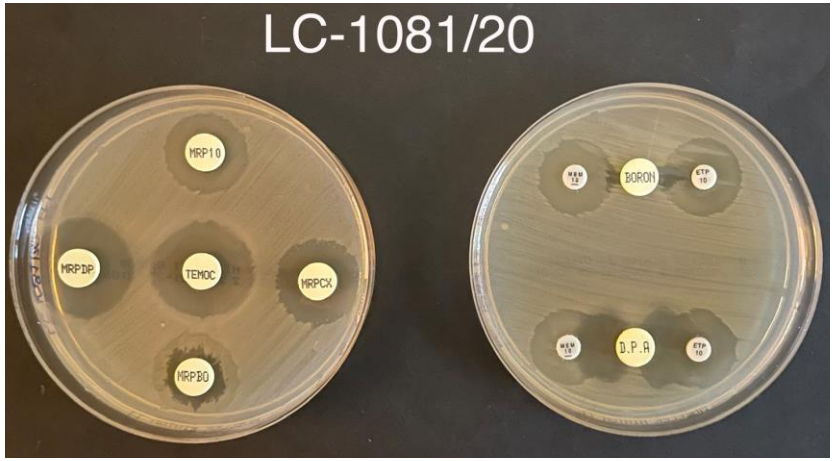

Proteus mirabilis RS

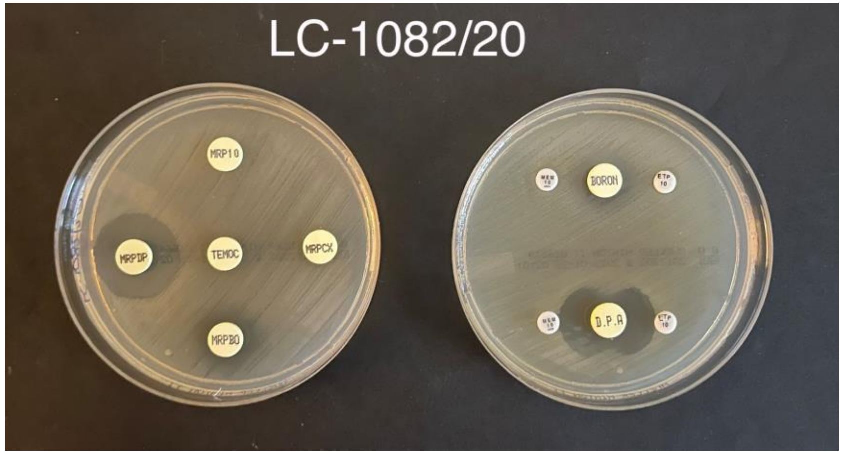

Enterobacter cloacae RS

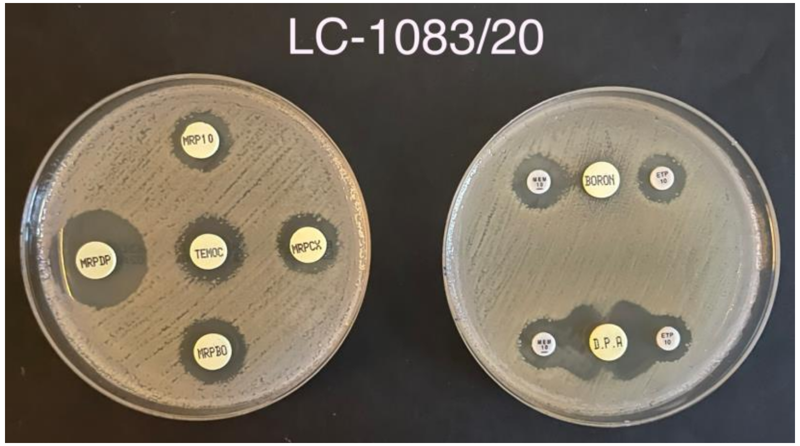

Klebsiella pneumoniae RS

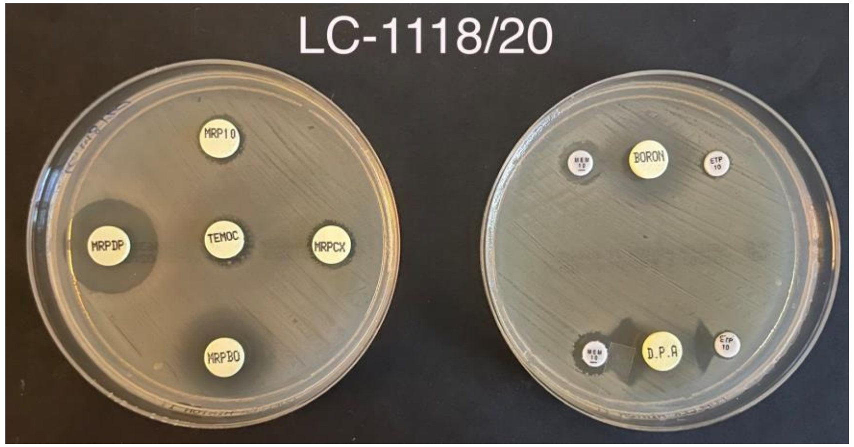

Enterobacter cloacae SW

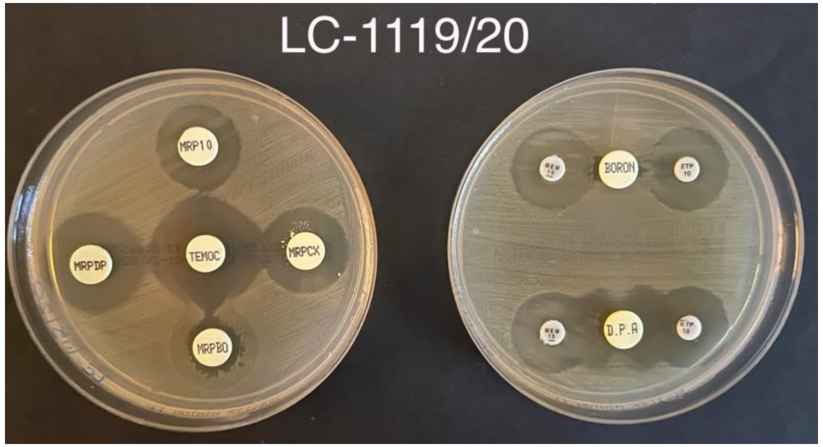

Proteus mirabilis SW

Figure S1. (b) Phenotypically evaluation was performed on the basis of synergistic activity between meropenem and dipicolinic acid using both the double disk approximation test (Rosco Diagnostica A/S, Taastrup, Denmark) and a commercially available combination method (KPC + MBL Confirm ID Kit; Rosco Diagnostica A/S).
